# PEGylated Gold Nanoparticles Grafted with N-Acetyl-L-Cysteine for Polymer Modification

**DOI:** 10.3390/nano11061434

**Published:** 2021-05-28

**Authors:** Dominik Fajstavr, Adéla Karasová, Alena Michalcová, Pavel Ulbrich, Nikola Slepičková Kasálková, Jakub Siegel, Václav Švorčík, Petr Slepička

**Affiliations:** 1 Department of Solid State Engineering, The University of Chemistry and Technology, 166 28 Prague, Czech Republic; dominik.fajstavr@vscht.cz (D.F.); adelakarasova@gmail.com (A.K.); nikola.kasalkova@vscht.cz (N.S.K.); jakub.siegel@vscht.cz (J.S.); vaclav.svorcik@vscht.cz (V.Š.); 2 The Department of Metals and Corrosion Engineering, The University of Chemistry and Technology, 166 28 Prague, Czech Republic; alena.michalcova@vscht.cz; 3 Department of Biochemistry and Microbiology, University of Chemistry and Technology Prague, 166 28 Prague, Czech Republic; pavel.ulbrich@vscht.cz

**Keywords:** nanoparticles, noble metal, sputtering, grafting, polymer, plasma modification

## Abstract

The subjects of this work were the enhancement and determination of the stability and other properties of gold nanoparticles (AuNPs) in an aqueous solution, gold nanoparticle immobilization, and further surface grafting on polyethylene naphthalate (PEN). Gold nanoparticles in PEG with a subsequent water solution addition were prepared using cathode sputtering; for the subsequent surface activation, two different solutions were used: (i) sodium citrate dihydrate (TCD) and (ii) N-acetyl-L-cysteine (NALC). The aim of this work was to study the effect of the concentration of these solutions on AuNPs stability, and further, the effect of the concentration of gold nanoparticles and their morphology, and to describe the aging process of solutions, namely, the optical properties of samples over 28 days. Stabilized AuNPs were prepared in an N-acetyl-L-cysteine (NALC) system and subsequently immobilized with NALC. The surface chemistry modification of AuNPs was confirmed using HRTEM/EDS. Gold nanoparticles were successfully immobilized with NALC. Grafting of the modified PEN from a solution of colloidal gold stabilized in the PEG–H_2_O–NALC system led to the polymer surface functionalization.

## 1. Introduction

Metallic colloidal nanoparticles can be prepared via top-down methods, which are usually physical methods, using bulk materials in a controlled process to form nanostructures/nanoparticles. These include molecular epitaxy, vacuum evaporation, or cathode sputtering [1,2,3]. In contrast, bottom-up methods, mainly wet chemical preparations of nanoparticles, are based on molecular aggregation, nucleation, and controlled growth of nanoclusters using donor ligands, polymers, and surfactants, which also serve as stabilizers. These include the chemical reduction of metal salts [4,5,6], electrochemical processes, or the controlled decomposition of metastable organometallic compounds [7].

For the first time, an innovative study was conducted to describe a versatile process for preparing magnetic colloidal solutions via sputtering on liquid surfaces [8]. It was shown for the first time that metallic colloidal NPs can be obtained by sputtering on silicone oil. However, silicone oil is not a good stabilizing agent and the obtained colloids were not stable. To avoid agglomeration, another liquid medium must be used. An ionic liquid (IL) seems to be the ideal type of liquid for this method of NP preparation. The sputtering of nanoparticles on ILs is a research area that began in 2006 with a groundbreaking study by Torimoto and co-authors [9]. Gold nanoparticles (AuNPs) with a diameter of about 5.5 nm were obtained by sputtering from an Au target in an ionic (Ar+) medium directly onto the surface of 1-ethyl-3-methylimidazolium tetrafluoroborate. Nanoparticles (silver nanoparticles) were sputtered into a liquid medium (silicone oil) in 1996 using radiofrequency magnetron deposition [10]. However, ILs are not the only liquid substrates used for sputtering metal nanoparticles. For example, vegetable oils, glycerol, or liquid polyethylene glycol are widely used for this method [11,12,13,14,15].

Electrical and optical properties depend on the structure of nanoparticles. Spherical AuNPs exhibit a variety of colors (e.g., brown, orange, red, and purple) in aqueous solution as their core size increases from 1–100 nm, and generally, AuNPs exhibit an absorption peak between 500–550 nm. AuNPs are considered to be one of the most suitable carrier systems due to their increased biocompatibility, stability, low toxicity, and oxidation resistance. Thus, colloidal gold (which may also be attached to a polymer surface) is useful in various medical disciplines, including biosensors [16,17] and biological detection, catalysis, bioelectronics, drug delivery and macromolecular carriers, and bioimaging [18]. PEG is a very weakly immunogenic substance, making it suitable for the development of PEG–protein conjugates as drugs [19,20]. Polyethylene glycol (PEG) modification, or PEGylation, is a common method of functionalizing gold nanoparticles. AuNPs are prepared simply via cathodic sputtering into liquid PEG as a capture medium without additional chemical reactions or added stabilizers. The stability and functional integrity of PEGylated AuNPs is dependent on many factors, including the size of the AuNPs used, the molecular weight of PEG, the attached functional groups, and the ligand used for PEGylation [21]. Nanoparticles can be coated with a layer of PEG alone or PEG in conjunction with other molecules, such as biotin, peptides, or oligonucleotides, to assist in the implementation of AuNPs into target cells. Due to their ability to bind cell membranes, these functionalized AuNPs can serve as good drug carriers. PEGylated AuNPs functionalized with biomolecules, such as lectin, lactose, and biotin, were synthesized in [22,23]. PEGylated AuNPs are useful in the cellular and intracellular targeting of biological materials. Hetero-bifunctional PEGylated AuNPs were synthesized in which AuNPs were functionalized with a thiol group at one end and coumarin, a fluorescent dye, on the other. These functionalized AuNPs could reach cells that could be easily monitored due to the attached dye [24].

Another effective way to increase the specificity and efficiency of nanoparticle systems is via functionalization with amino acids and peptides. AuNPs conjugated to amino acids, such as lysine, polylysine, and glycine, bind DNA with greater efficiency to gene transport without toxicity. The primary ammonium groups of these amino acids contributed to a higher binding ability of cationic groups to DNA [25]. Amino-acid-functionalized gold colloids provide a backbone for efficient DNA binding. The amino-acid-based NPs responded to intracellular levels of glutathione and provided a tool for controlled release and concomitant DNA expression [26]. In his work [27], Russier-Antoine described a simple synthesis that produces large chiral supramolecular nanocomplexes of gold and cysteine. Gold–cysteine polymers of NP induced remarkable two-photon nonlinear spectra and so-called hyper-polarizability. Thus, they appear to be good candidates for nonlinear optical microscopy. The high arginine peptide (CLANNR8) was conjugated to AuNPs for transport into a cell membrane [28]. Some research groups have developed methods for functionalizing gold nanoparticles and other nanoparticles using oligonucleotides either alone or with some modifications. DNA-conjugated nanostructures can be synthesized in a controlled manner, either by attaching a specific number of single-stranded DNA molecules via thiol caps or by saturating the surface of gold nanoparticles with single-stranded DNA molecules [29]. Kinetic and thermodynamic studies of DNA hybridized to AuNP have shown that DNA first adheres to nanoparticles and then slowly diffuses to its surface [30]. Aptamer–AuNP conjugation was recently used to target prostate cancer cells [31]. This was accomplished by attaching AuNP to an oligonucleotide complementary to the anti-PSMA (prostate-specific membrane antigen) sequence, which facilitates the attachment of PSMA–AuNP to the anti-PSMA antibody. In summary, the most commonly used functionalization methods involve application of the following functional groups: PEG [32,33,34,35], amine group [36], carboxyl group [37], peptide [38,39], or DNA [40,41]. For applications in tissue engineering for the support of cell growth or as antibacterial properties, the as-sputtered nanoparticles in water solution or grafted nanoparticles on polymer substrates were used recently [42,43,44,45].

AuNPs were prepared via cathode sputtering into PEG, followed by transfer into solutions of sodium citrate dihydrate or N-acetyl-L-cysteine (LNAC). The effects of the concentrations of these solutions on the stability of AuNPs were studied. In addition, the concentration of nanoparticles, their morphology, and the change in optical properties of colloidal solutions during aging were monitored. Sample analysis was performed using UV-Vis spectroscopy, atomic absorption spectrometry (AAS), transmission electron microscopy (TEM), and high-resolution TEM (HRTEM). The described AuNP/NALC/H_2_O/PEG solutions were then used to graft the surface of polyethylene naphthalate. To our best knowledge, the combination of PEN plasma treatment for its activation combined with subsequent grafting in a colloidal gold solution for gold grafting has never been published. It was shown to successfully functionalize the polymer surface with AuNPs. 

## 2. Materials and Methods

### 2.1. Materials

Colloidal gold was obtained via cathodic sputtering from a Au target (Safina s.r.o., purity 99.9999%, Vestec, Czech republic) into a liquid medium. PEG-600 (polyethylene glycol with an average molecular mass of 600 Da, Sigma-Aldrich, St. Louis, MO, USA) was selected as the medium. PEG is a hygroscopic colorless viscous liquid at room temperature, is fully water-soluble, and is miscible with water in all proportions. PEG is a commonly used cathode sputtering medium because it has a sufficiently low saturated vapor pressure at deposition pressures, stabilizes nanoparticles without the need for additional compounds, and is a biocompatible material with low toxicity [1].

**Figure 1:**
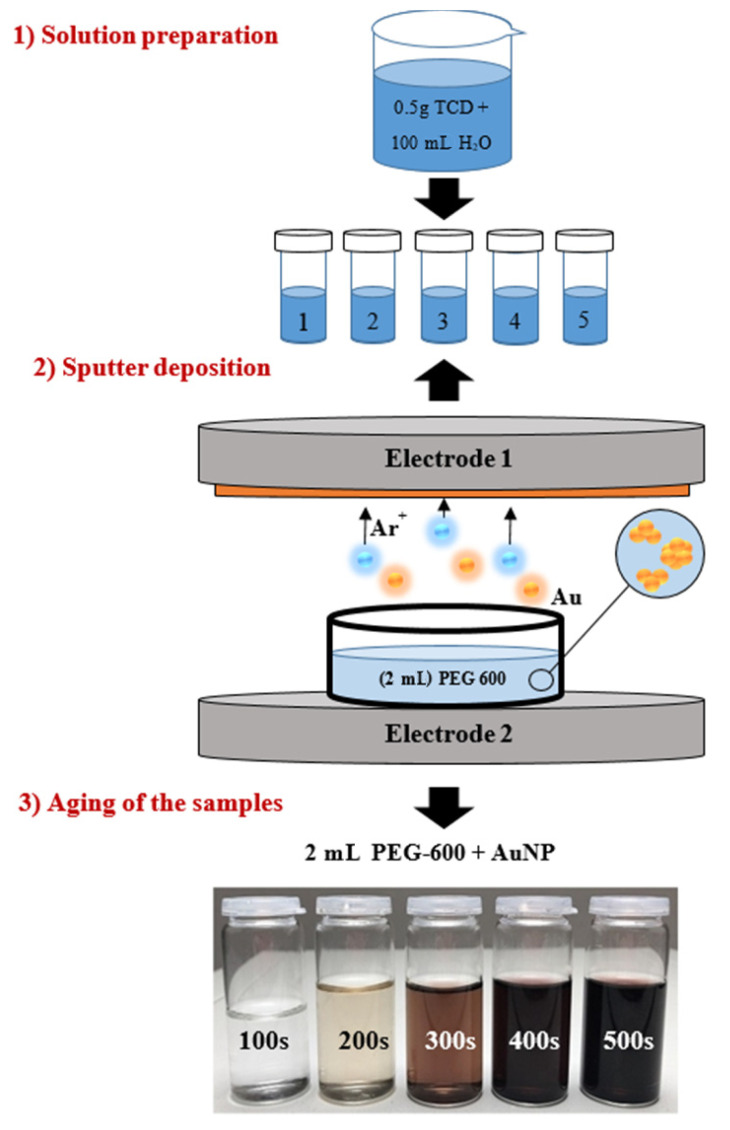
Principle of the preparation of a series of AuNPs solutions for one concentration of TCD (*c* = 17.0 mmol∙L^−1^): (**1**) preparation of the TCD solution and subsequent dosing of 18 mL into glass sample containers; (**2**) deposition of AuNPs into 2 mL PEG-600 at selected times (100–500 s), followed by the addition of TCD; (**3**) after aging of the products.

Sputtering was carried out at 20 °C in a Sputter Coater SCD 050 (Baltec, Balzers, Liechtenstein) at an argon pressure of approximately 8 Pa, a current of 30 mA, and an electrode gap of 50 mm. Immediately after the deposition of the AuNPs into PEG-600, the mixture was diluted to an aqueous solution in a PEG:water ratio of 1:9. Either an aqueous solution of sodium citrate dihydrate (TCD—a white crystalline powder, *M*_w_ = 294.10 g·mol^−1^, Sigma Aldrich, ) or the amino acid solution N-acetyl-L-cysteine (NALC—white crystalline solid, *M*_w_ = 163.19 g·mol^−1^, Sigma Aldrich) was used to modify the nanoparticles. The stability of the solutions and the size of the nanoparticles were monitored such as the dependence of this factors on the amount of TCD or amino acid in the solution. TCD solutions were prepared at four concentrations as follows: (a) 17.0 mmol∙L^−1^ (by mixing 0.5 g TCD + 100 mL H_2_O), (b) 34.0 mmol∙L^−1^ (1.0 g TCD), (c) 51.0 mmol∙L^−1^ (1.5 g TCD), and (d) 68.0 mmol∙L^−1^ (2.0 g TCD). The sample preparation procedure was the same as for the TCD samples, but the deposition times (100, 300, 600, 900 s) and concentrations of the NALC solutions varied: (a) 30.6 mmol∙L^−1^ (by mixing 0.5 g NALC + 100 mL H_2_O), (b) 61.3 mmol∙L^−1^ (1.0 g NALC), and (c) 91.9 mmol∙L^−1^ (1.5 g NALC). A demonstration of the experiment is shown in Figure 1.

The last part of this work was focused on the study and modification of the surface of polyethylene naphthalate (PEN). Biaxially oriented PEN (density 1.36 g·cm^−3^, thickness 50 µm, *T*_m_ ~ 250–290 °C, *T*_g_ ~ 120 °C, supplied by Goodfellow Ltd., Cambridge Ltd., Huntington, UK) was used. 

The plasma modification method was chosen for the PEN surface activation. Balzers SCD 050 from BAL-TEC was used and the etching mode was applied. The modification conditions were as follows: temperature 20 °C, pressure 8 Pa, and modification power of 8 W. Modification was carried out for the following deposition times: 200, 400, 600, 800, and 1000 s.

For the next experiment, a solution of AuNPs in PEG/H_2_O/NALC at an NALC/H_2_O ratio of 1.5 g/100 mL was prepared. PEN samples that were modified by plasma exposure for 200 and 400 s were immersed in a colloidal gold solution for 24 h. After 24 h, the PEN samples were removed from the solution, washed with distilled water, and dried at room temperature. The PEN samples thus prepared were subjected to AFM (Bruker Corp., Billerica, MA, USA) and XPS (Scienta Omicron GmbH, Taunusstein, Germany) analysis. Changes to the PEN morphology after grafting to the Au nanoparticles or the amino acid NALC were investigated in combination with a detailed analysis of the chemistry of the grafted surface.

### 2.2. Characterization Techniques

Samples were weighed using the Metler Toledo UMX2 automatic microbalance (Mettler-Toledo, Columbus, GA, USA). UV-Vis spectroscopy was used to characterize the optical properties of the samples. The absorbance of AuNPs solutions was measured in a 1 cm glass cuvette using a Perkin-Elmer spectrophotometer Lambda 25 (Waltham, MA, USA). Spectra were recorded at room temperature in the range of 250 to 800 nm. Gold nanoparticles with a spherical shape exhibited only one peak at 520 nm at this interface. This optical property changes with the AuNPs shape.

The determination of the gold concentration in the solutions was performed on an Agilent 280FS AAS spectroscope (Santa Clara, CA, USA) with a flame atomization technique. The determination was carried out by flame atomization (acetylene–air) at a wavelength of 242.8 nm. Atomic absorption spectrometry (AAS, Agilent, Santa Clara, CA, USA) is a spectrometric analytical method for the determination of trace and significant concentrations of individual elements in the analyzed solution. It utilizes the absorption of radiation by free atoms of the monitored element. For the generation of free atoms, a flame is most often used in the AAS.

Transmission electron microscopy (TEM) was used to monitor and study the size and shape of nanoparticles in solutions. TEM samples were analyzed on a JEOL JEM-1010 transmission electron microscope. The images were taken with an SIS Megaview III digital camera (Soft Imaging Systems, 80 kV acceleration voltage, Tokyo, Japan), and analysis was performed with AnalySIS 2.0 software (Tokyo, Japan). High-resolution transmission electron microscopy (HRTEM) was used to characterize the solutions in more detail. An EFTEM Jeol 2200 FS (Tokyo, Japan) with a dot resolution of 0.23 nm was used. A transmission electron microscope equipped with an energy filter enabled operation at accelerating voltages up to 200 kV. HRTEM analysis was used in conjunction with electron dispersion spectroscopy (EDS, Joel Ltd., Tokyo, Japan). The resolution of the EDS analysis was 1–2.4 nm. The chemical compositions of the samples were measured using EDS. Some solutions were subjected to EDS element mapping and point analysis.

Atomic force microscope (AFM Dimension ICON Bruker Corp., Billerica, MA, USA) in QNM mode was used to study the surface morphology of the PEN layer in an ambient atmosphere. The silicon tip was mounted on a SCANASYST-AIR nitride cantilever with a spring constant of 0.4 N·m^−1^. Data were processed using NanoScope Analysis software (Billerica, MA, USA).

The quantitative composition of the PEN surface was obtained using X-ray photoelectron spectroscopy (XPS, Scienta Omicron GmbH, Taunusstein, Germany). An ESCAProbeP spectrometer manufactured by Omicron Nanotechnology Ltd. (East Grinstead, UK) was used for the measurements. The sample area analyzed was 3 × 4 mm2. The surface of the sample was excited using X-rays from a 1488.7 eV monochromatic source. The relative representation of elements was evaluated from the peak intensities in the XPS spectra using CasaXPS software (2.3.1, Wilmslow, UK).

## 3. Results

### 3.1. Stability Studies of AuNP Solutions in Sodium Citrate Dihydrate

Evidence of the presence of gold nanoparticles was obtained from the UV-Vis spectra. The peak height provides information on the concentration of the AuNPs in the solution. According to the peak width, it is possible to conclude information about the stabilization of homogeneous solutions. Shifts to the longer wavelengths are typical of increasing NP sizes. According to the shapes of the peaks, these were all spherical nanoparticles.

**Figure 2:**
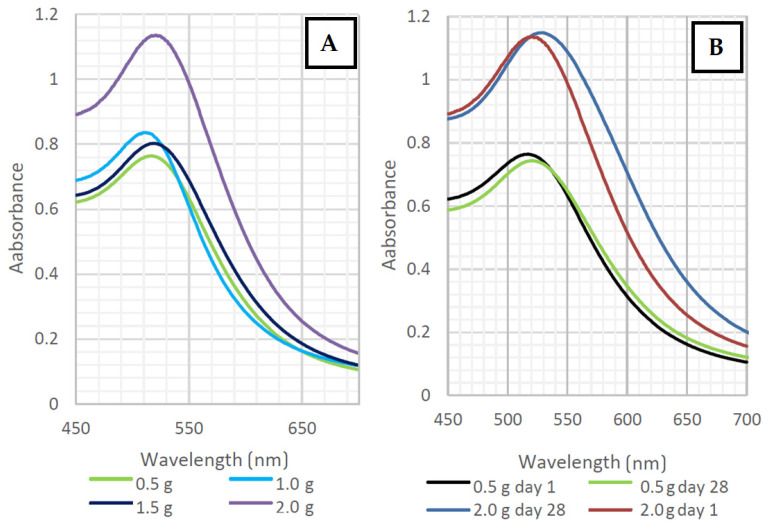
(**A**) Changes in the absorbances of AuNPs in PEG/TCD/H_2_O with different TCD/H_2_O ratios (0.5 g/100 mL, 1.0 g/100 mL, 1.5 g/100 mL, and 2.0 g/100 mL) prepared with a deposition time of 300 s; (**B**) changes in the AuNPs’ absorbances in PEG/TCD/H_2_O with different TCD/H_2_O ratios (0.5 g/100 mL and 2.0 g/100 mL) prepared with a deposition time of 300 s during the aging process.

Colloidal solutions of prepared AuNPs that were stabilized with distilled water with TCD showed absorption peaks of UV-Vis spectra. In Figure 2A, we can see that the absorbance was around 0.8 for all samples, except for the sample with the highest TCD concentration (2 g/100 mL).

**Figure 3:**
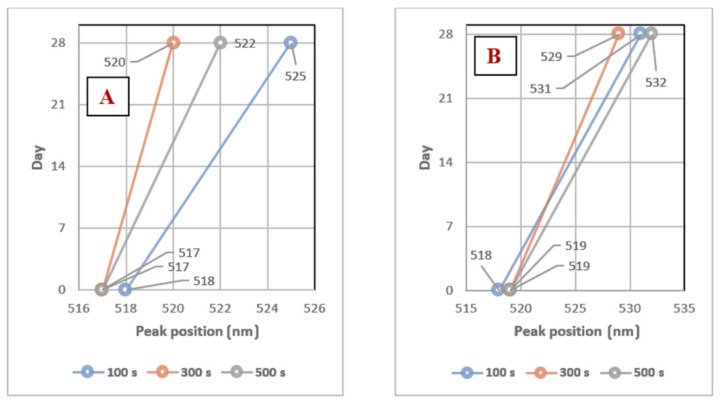
(**A**) AuNPs absorption peak positions in PEG/TCD/H_2_O with a TCD/H_2_O ratio of 0.5 g/100 mL prepared with deposition times of 100, 300, and 500 s; (**B**) AuNPs’ absorption peak positions in PEG/TCD/H_2_O with a TCD/H_2_O ratio of 1.5 g/100 mL prepared with deposition times of 100, 300, and 500 s.

It is apparent from both graphs (Figure 2A,B) that the changes in the TCD/H_2_O ratio had no significant effect on the distribution of the AuNPs sizes. In Figure 2B, we can observe the change in size, shift, and broadening of the absorption peaks, indicating a slight aggregation and growth of the nanoparticles during aging. The high absorbance value of AuNPs in the TCD/H_2_O solution at a ratio of 2 g/100 mL may have been caused by the higher concentration of TCD in the solution. Peak positions were in the range of 516–532 nm. The peak shifts during the 28-day aging are shown in the following graphs in Figure 3.

**Figure 4:**
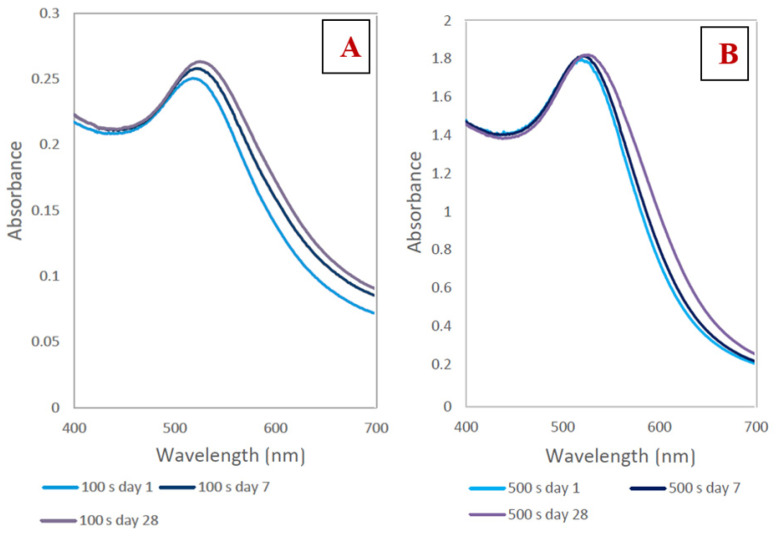
(**A**) Absorbance of AuNPs in PEG/TCD/H_2_O with a TCD/H_2_O ratio of 0.5 g/100 mL prepared with a deposition time of 100 s during the aging process; (**B**) absorbance of AuNPs in PEG/TCD/H_2_O with a TCD/H_2_O ratio of 2 g/100 mL prepared with a deposition time of 500 s during the aging process.

Solutions prepared with a TCD/H_2_O ratio of 0.5 g/100 mL demonstrated high stability, with the absorption peak shifted by only 4–7 nm over 28 days. For a solution with a higher TCD/H_2_O ratio (1.5 g/100 mL), the absorption peaks shifted between 10–13 nm. For this reason, both solutions could be considered stable, but higher concentrations of TCD had a greater influence on the optical properties of the solutions during the aging process. In this work, many different combinations of solutions (TCD/H_2_O) were made; therefore, only two samples were chosen for further comparison of the AuNPs stability in TCD/H_2_O/PEG solutions, which differed the most in both the deposition time and TCD/H_2_O ratio. The comparisons of the UV-Vis solution spectra are shown in Figure 4A,B.

The UV-Vis spectra peak maximum for both samples shown in Figure 4A,B ranged between 513 and 532 nm. We managed to obtain stable AuNPs in all solutions. However, these two solutions differed significantly in terms of their absorption maxima. A shorter deposition time (100 s, Figure 4A) resulted in a lower concentration of AuNPs in the solution. Longer deposition times (500 s, Figure 4B) led to a higher content of nanoparticles in solution and an increase in absorbance.

**Figure 5:**
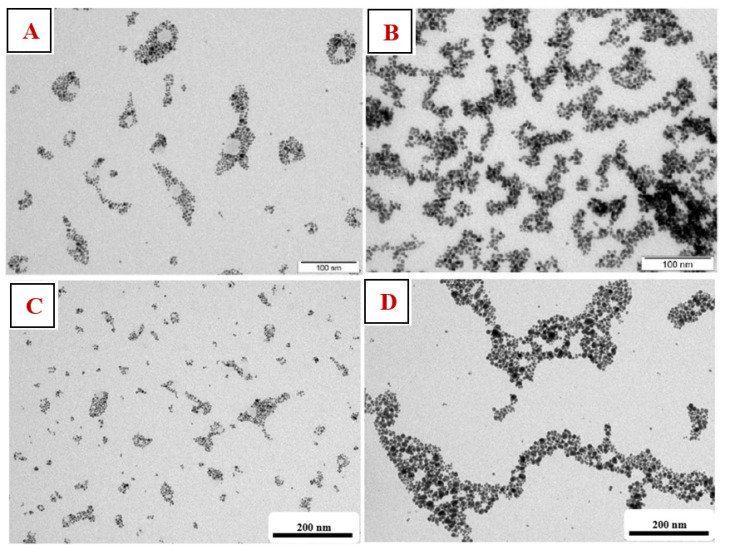
TEM AuNP images in PEG/TCD/H_2_O prepared with a deposition time of 300 s. (**A**) TCD/H_2_O (0.5 g/100 mL) solution and (**B**) TCD/H_2_O (1.5 g/100 mL) solution. TEM AuNP images in PEG/TCD/H_2_O at a TCD/H_2_O ratio of 0.5 g/100 mL prepared with deposition times of 100 (**C**) and 500 s (**D**).

Selected samples were studied using TEM analysis. In Figure 5, we can see that the prepared AuNPs had a spherical shape. From the images of Figure 5C,D, it can be seen that longer deposition times led to more concentrated solutions and larger particles; AuNP agglomerates of the ‘chain’ type were up to 200 nm in size. A solution with a higher concentration of TCD formed a denser AuNPs network due to the affinity of AuNPs for the TCD that the nanoparticles enveloped.

### 3.2. Study of AuNP Stability in N-Acetyl-L-Cysteine

The prepared AuNPs solutions stabilized in NALC were analyzed immediately after preparation and the UV-Vis spectra of the prepared AuNPs solutions were obtained. The UV-Vis spectra of the solutions with NALC with a deposition time of 100 and 300 s showed a broad band with no absorption maximum. The presence of colloidal gold in these solutions was not confirmed using this method. For solutions with higher deposition times (600 and 900 s), the positions of the absorption maxima ranged between 459 and 515 nm, but the peaks had a very flat profile. The solution with the highest concentration of NALC showed the most obvious absorption maxima (1.5 g NALC/100 mL H_2_O), where the absorption maxima ranged from 513 to 515 nm (for 600 and 900 s), but the curve profiles were still very wide. With higher deposition times, the absorbance of the solutions also increased. 

**Figure 6:**
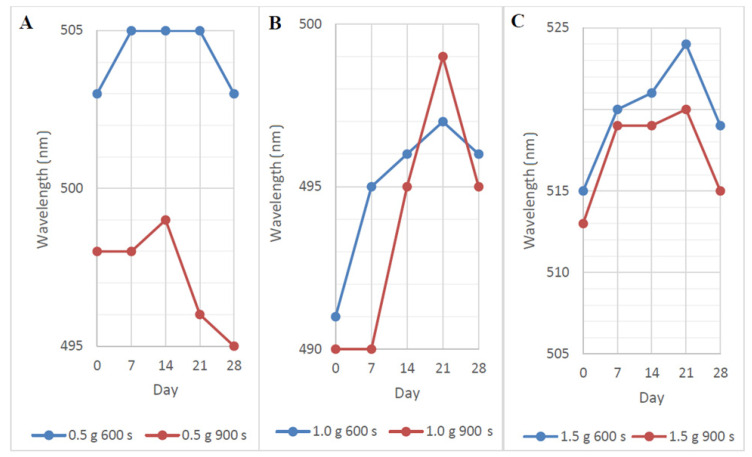
AuNP absorption peak positions in PEG/NALC/H_2_O prepared with a deposition time of 600 or 900 s in NALC/H_2_O: (**A**) 0.5 g/100 mL, (**B**) 1.0 g/100 mL, and (**C**) 1.5 g/100 mL.

The solutions described in Figure 6 showed slightly different absorbances during the aging process. The positions of the absorption peaks shifted by 0–5 nm over 28 days, indicating their good stability. By day 2, the peak position was red-shifted, indicating possible aggregation of the particles. After the 21st day, the values started to fall again, and AuNP could already be degraded. The peak positions for NALC/H_2_O 0.5 g/100 mL solutions ranged from 498 to 503 nm; for the solutions with 1.0 g/100 mL, the positions ranged from 490 to 496 nm; for the solutions with 1.5 g/100 mL, the positions ranged from 513 to 519 nm (Figure 6).

**Figure 7:**
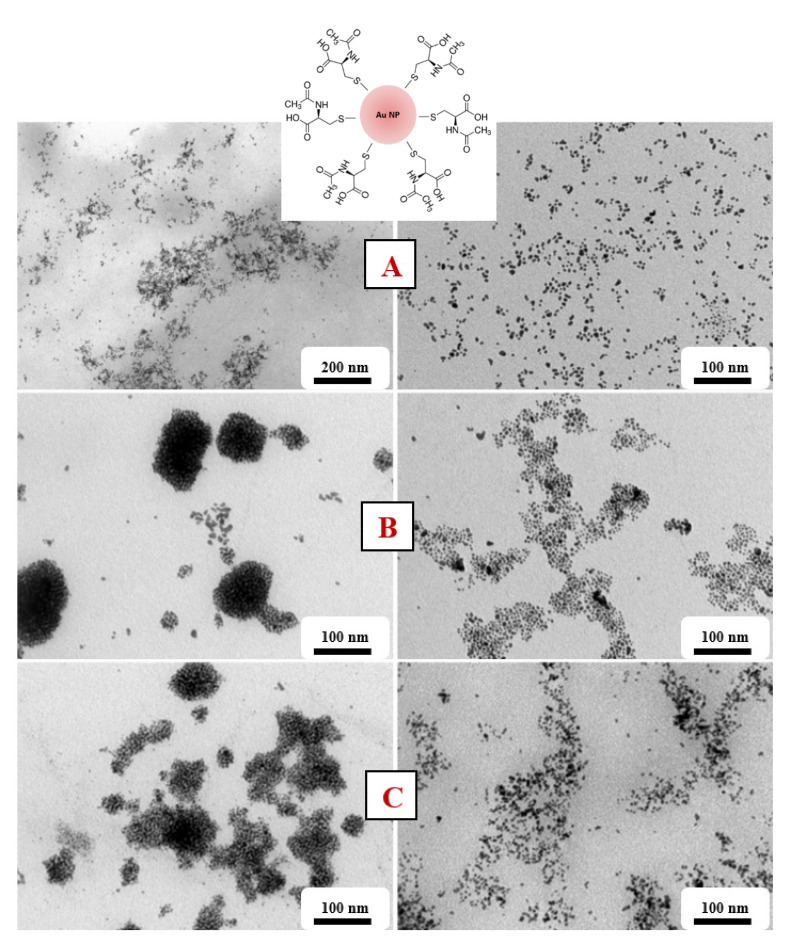
TEM AuNP images in PEG/NALC/H_2_O prepared with a deposition time of 900 s. (**A**) NALC/H_2_O solution (0.5 g/100 mL), (**B**) TCD/H_2_O solution (1.0 g/100 mL), and (**C**) TCD/H_2_O solution (1.5 g/100 mL). Right column after washing with distilled water. A scheme of a grafted Au nanoparticle with NALC is also introduced.

From the TEM images (Figure 7), we can see that we managed to create spherical AuNPs independent of the concentration of NALC/H_2_O in the solutions. However, solutions with a higher proportion of NALC (1.0 and 1.5 g/100 mL H_2_O) showed significant agglomerations of gold nanoparticles. NALC probably wrapped individual AuNPs and together formed a core–shell structure of Au–NALC. This increased their affinity for other non-enveloped AuNPs and clustering could occur. After washing these samples with distilled water, the clusters were no longer so apparent; there was probably a partial ‘washing’ of the coating. This may have also been affected by the existence of weak van der Waals interactions between the particles such that washing the particles may have affected the interactions between them.

**Figure 8:**
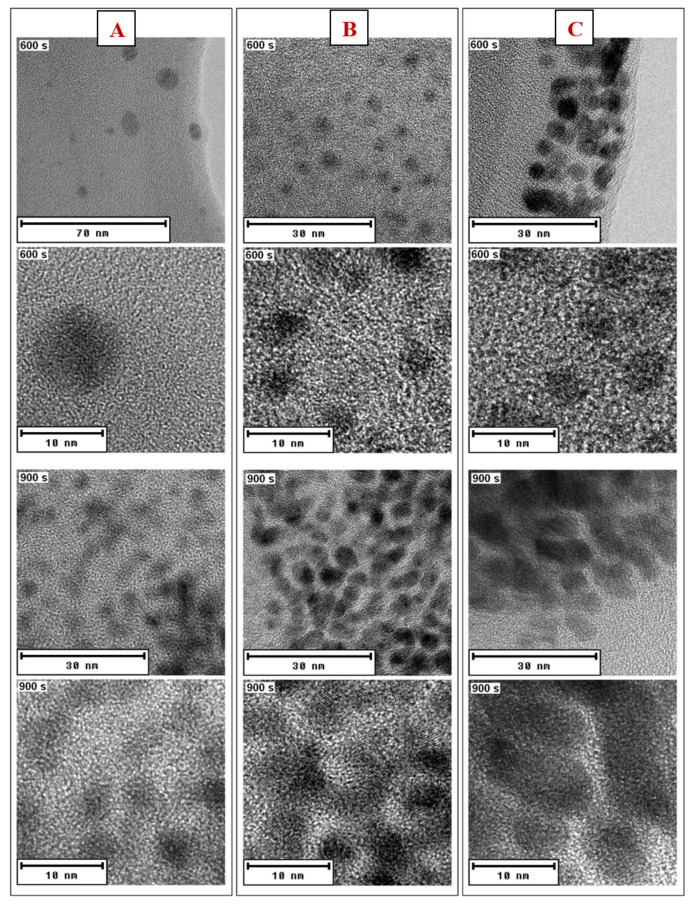
HRTEM AuNP images in PEG/NALC/H_2_O prepared with a deposition time of 600 or 900 s: (**A**) NALC/H_2_O solution (0.5 g/100 mL), (**B**) TCD/H_2_O solution (1.0 g/100 mL), and (**C**) TCD/H_2_O solution (1.5 g/100 mL).

**Figure 9:**
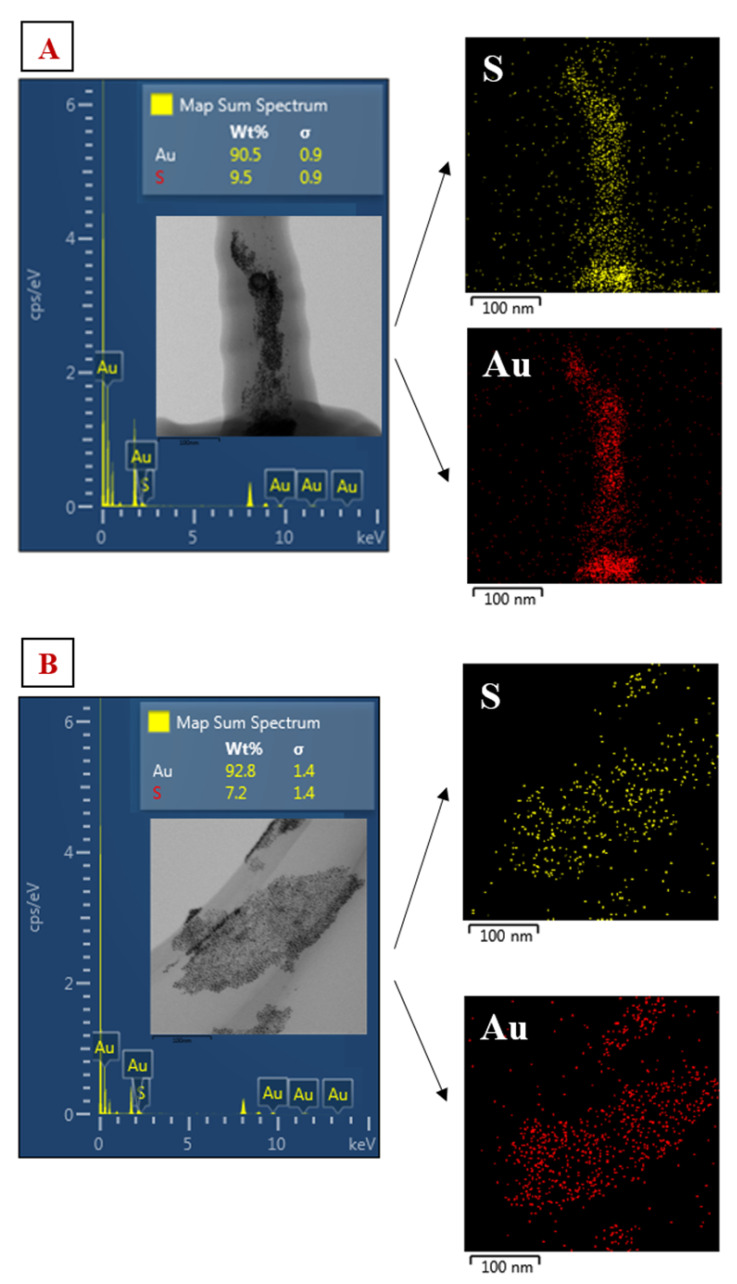
(**A**) Sample analysis of AuNP-900 s/NALC/H_2_O at an NALC/H_2_O ratio of 0.5 g/100 mL; EDS spectrum—Au and S in the sample. HRTEM image of the sample, EDS mapping of Au and S content in solution. (**B**) Analysis of AuNP-600 s/NALC/H_2_O sample at an NALC/H_2_O ratio of 0.5 g/100 mL; EDS spectrum—Au and S in the sample. HRTEM image of the sample, EDS mapping of Au and S content in solution.

We managed to obtain spherical gold nanoparticles with sizes in the range of 6–10 nm (Figure 8). The concentration of the amino acid in the solution did not affect the size of the generated AuNPs, only their aggregation. EDS analysis confirmed the presence of Au and S in the solutions (Figure 9).

### 3.3. Modification of PEN Using AuNP/PEG/NALC/H_2_O

PEN was modified using plasma with exposure times of 200–1000 s. Ablation times of 200 and 400 s were chosen for grafting PEN foils since these plasma modification times are sufficient to activate the surface of the material based on our previous experiments. After the PEN activation, samples were exposed to the AuNP/PEG/NALC/H_2_O solution for 24 h, followed by changes in the PEN morphology and chemistry.

**Figure 10:**
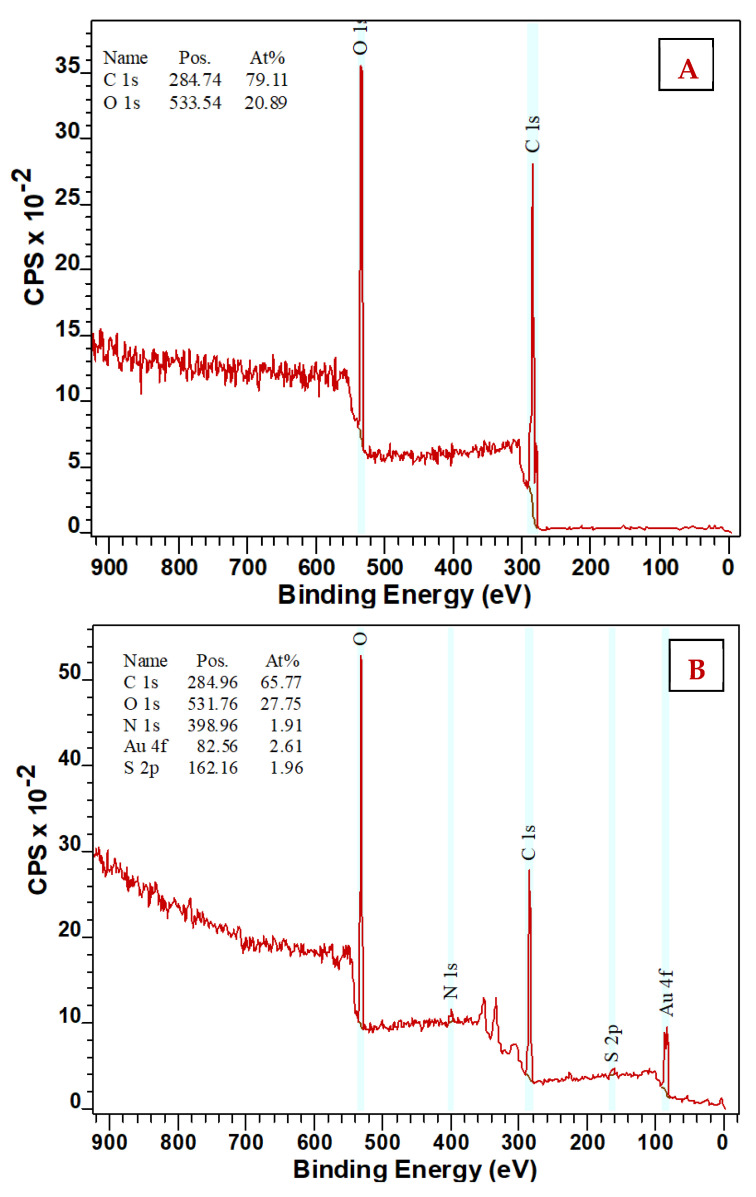
XPS analysis: (**A**) non-modified PEN and (**B**) PEN plasma modified for 400 s after 24 h immersion in AuNP/PEG/NALC/H_2_O solution (NALC/H_2_O ratio-1.5 g/100 mL, AuNP deposition time-600 s).

Changes in the chemical composition of the surface layer were studied using the XPS method (Figure 10). XPS analysis showed the presence of sulfur (S) and gold on the PEN surface for all studied samples. The values of the individual atomic concentrations of sulfur and gold are demonstrated in Table 1 below. Since the original PEN did not contain gold or sulfur, the results show that the immersion of PEN samples in AuNP/NALC solutions bound to AuNP–NALC in the polymer surface layer.

The concentrations of bound gold and sulfur were the highest for the 400 s plasma-modified sample that was immersed in the AuNP solution prepared with a deposition time of 600 s. In the previous part of this work, it was discussed that a high concentration of NALC in solution may cause AuNPs amino acid ‘wrapping’ or ‘chaining,’ which was also probably the reason for lower gold concentration values on the PEN surface. 

**Figure 11:**
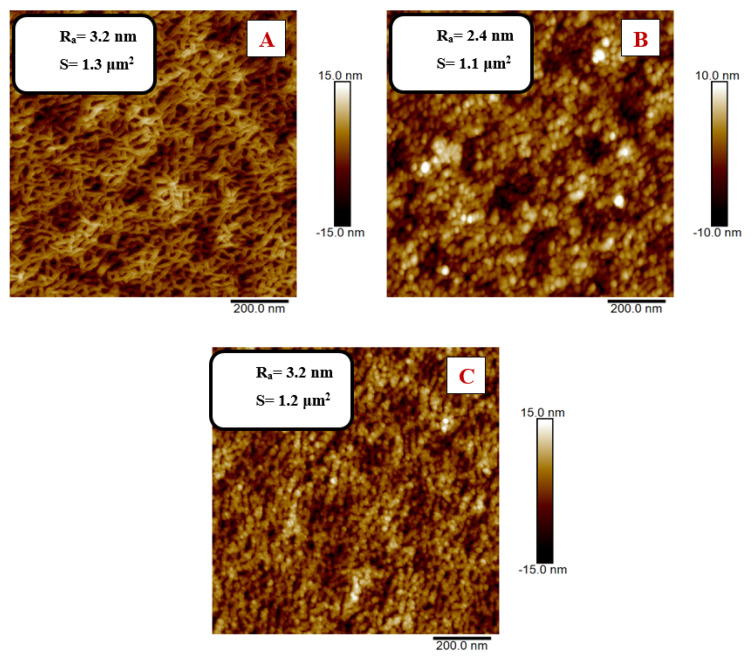
Two-dimensional AFM images of PEN that was modified using plasma for 200 s at 8 W power: (**A**) non-grafted sample; (**B**) grafted in AuNP/NALC (1.5 g)/H_2_O (100 mL)/PEG solution, AuNP deposition time 600 s; (**C**) grafted in AuNP/NALC (1.5 g)/H_2_O (100 mL)/PEG solution, AuNP deposition time 900 s.

**Figure 12:**
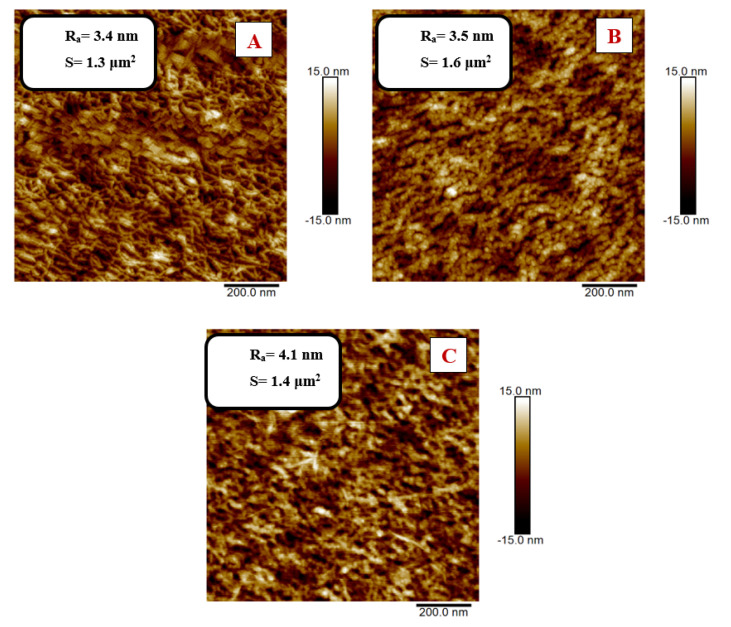
Two-dimensional AFM images of PEN that was modified using plasma for 400 s at 8 W power: (**A**) non-grafted sample; (**B**) grafted in AuNP/NALC (1.5 g)/H_2_O (100 mL)/PEG solution, AuNP deposition time 600 s; (**C**) grafted in AuNP/NALC (1.5 g)/H_2_O (100 mL)/PEG solution, AuNP deposition time 900 s.

The binding of elements to the polymer surface was also reflected in the change in its surface morphology, which was studied using AFM. In Figure 11 and Figure 12, there are 2D images of the surface of the modified PEN before and after the Au/amino acid grafting. At the same time, the surface roughness values R_a_ and effective area S are presented. It is apparent from the AFM results that the surface morphology changed due to grafting.

In Figure 11, we can see that after the plasma modification for 200 s, the surface of the PEN had a lamellar structure; after grafting, a globular structure appeared on the surface of the PEN with a significant increase in the surface segmentation. The globular structure results from the interaction of PEN with the solution, but mainly from the binding of Au, which was confirmed by the abovementioned XPS analysis. Furthermore, a decrease in the effective area S after grafting in solution with AuNP deposited at 600 s and a subsequent increase in S after grafting in solution with AuNP deposited at 900 s are shown.

As can be seen in Figure 12, after the 400 s plasma modification, the PEN surface had a more structured lamellar structure; after grafting, globular structures appeared on the activated PEN surface. The change in effective area S had the same trend as for the 200 s modified PEN. The grafting in the AuNP-600 s solution led to a decrease in S, while the grafting in the AuNP-900 s solution increased the effective area.

In both cases (200 and 400 s), a significant change in the surface roughness is visible in the images compared to the AuNP-600 s and AuNP-900 s. This change in R_a_ was due to the formation of AuNP aggregates due to the higher concentration of AuNP in the solution.

## 4. Conclusions

Gold nanoparticles were prepared via sputter deposition into polyethylene glycol. AuNPs were stabilized in the PEG–H_2_O–TCD system. Spherical AuNPs of sizes up to 10 nm were formed. Higher sodium citrate concentrations in solutions were found to have a significant effect on the optical properties of the colloidal solutions. In the PEG–H_2_O–TCD system, AuNPs began to cluster into visible structures in as early as 14 days. This additional aggregation was probably due to the coating and agglomeration of unreacted citrate particles.

AuNPs were also stabilized in the PEG–H_2_O–NALC system. We managed to prepare spherical grafted Au/NALC nanoparticles with sizes up to 10 nm. Samples with higher AuNP deposition times showed higher stability. However, the AuNP size increased during the aging of the solutions. Higher concentrations of NALC in solutions led to the aggregation of nanoparticles and even ‘chaining.’ Otherwise, the concentration of NALC in the solutions did not affect the size of the formed nanoparticles.

Grafting of the modified PEN from a solution of colloidal gold stabilized in the PEG–H_2_O–NALC system led to the polymer surface functionalization. XPS and AFM analyses confirmed the presence of Au in the PEN surface layer. The grafting of gold nanoparticles onto the polymer had a significant effect on the surface morphology of PEN when the original linear structure changed to a globular structure.

## Figures and Tables

**Table 1:** Atomic concentrations of elements (S, Au) determined using the XPS method in pristine PEN, PEN modified with plasma 8 W for 200 and 400 s, and PEN modified with plasma 200 and 400 s after 24 h of immersion in AuNP/NALC/H_2_O solution (NALC/H_2_O ratio—1.5 g/100 mL), deposition time 600 and 900 s.

Sample	Element Concentration (at%)	
	S	Au
Pristin PEN; PEN modif. 200 s; PEN modif. 400 s	-	-
PEN modif. 200 s, AuNP 600 s	1.26	0.12
PEN modif. 200 s, AuNP 900 s	1.26	2.32
PEN modif. 400 s, AuNP 600 s	1.96	2.61
PEN modif. 400 s, AuNP 900 s	0.83	0.59

## Data Availability

Data are contained within the article.
